# Adaptations and modifications to a co-designed intervention and its clinical implementation: a qualitative study in Denmark

**DOI:** 10.1186/s12913-021-07142-4

**Published:** 2021-10-16

**Authors:** Jeanette Wassar Kirk, Per Nilsen, Ove Andersen, Nina Thórný Stefánsdóttir, Birk Grønfeldt, Rasmus Brødsgaard, Britt Stævnsbo Pedersen, Thomas Bandholm, Tine Tjørnhøj-Thomsen, Mette Merete Pedersen

**Affiliations:** 1 Present Address: Department of Clinical Research, Copenhagen University Hospital, Amager and Hvidovre, Kettegaard alle 30, 2650, Hvidovre, Danmark; 2 Department of Public Health, Nursing, Aarhus University, Nordre Ringgade 1, 8000, Aarhus, Denmark; 3 Department of Health, Medical and Caring Sciences, Linköping University, Sandbäcksgatan 7, 582 25 Linköping, Sweden; 4 Copenhagen Neuromuscular Center, Rigshospitalet, Inge Lehmanns Vej 8, 2100 Copenhagen Ø, Denmark; 5 Department of Orthopedic Surgery, and Department of Physical and Occupational Therapy, Physical Medicine & Rehabilitation Research-Copenhagen (PMR-C), Copenhagen University Hospital, Amager and Hvidovre, Kettegaards alle 30, 2650 Hvidovre, Denmark; 6 Department of Health and Social Context, National Institute of Public Health, University of Southern Denmark, Studiestræde 6, 1455 Copenhagen K, Denmark; 7 Department of Clinical Medicine, University of Copenhagen, Blegdamsvej 3B, 2200 Copenhagen N, Denmark

**Keywords:** Adaptation, Modification, Evidence-based interventions, Qualitative methods, Non-conscious processes

## Abstract

**Background:**

There is a long-standing debate in implementation research on whether adaptations to evidence-based interventions (EBIs) are desirable in health care. If an intervention is adapted and not delivered as conceived and planned, it is said to have low fidelity. The WALK-Cph project was developed based on the assumption that involving stakeholders in co-design processes would facilitate the fidelity of an intervention to increase the mobility of acutely admitted older medical patients and its implementation in two hospitals in Denmark. The purpose of this study is to describe and analyse adaptations and modifications that were made to the co-designed WALK-Cph intervention and its implementation.

**Methods:**

This study used a qualitative design. An ethnographic field study was performed using participant observations, workshops and semi-structured interviews. Data were analysed twice using the Framework Method. The first analysis was based on the frameworks from Stirman, Moore and Proctor. The second analysis, a retrospective modifications analysis, was based on the Adaptation-Impact Framework.

**Results:**

Many different types of adaptations and modifications were made to the WALK-Cph intervention and its implementation plan. Most of the modifications were made on the contents of the intervention. In total, 44 adaptations and modifications were made, of which 21 were planned (adaptations) and 23 were made haphazardly (modifications). Most of the content and context adaptations and modifications made on the intervention had a mixed result regarding enhanced fidelity. The retrospective modifications analysis showed that modifications were ongoing and both situationally and contextually shaped.

**Conclusions:**

Although an extensive co-design process was carried out to facilitate the fidelity of the WALK-Cph intervention, this study showed that many adaptations and modifications were still made to both the intervention and its implementation plan. It could indicate that the co-design process had a small effect or that adaptations and modifications are ongoing and both situationally and contextually shaped, which challenge the assumption and the desire to be able to plan and control changes.

**Supplementary Information:**

The online version contains supplementary material available at 10.1186/s12913-021-07142-4.

## Background

There is a long-standing debate in implementation research on whether adaptations to evidence-based interventions (EBIs) are desirable in health care [1]. If an intervention is adapted and not delivered as conceived and planned, it is said to have low fidelity [2]. Fidelity refers to the degree to which an intervention or programme is delivered as intended [3]. Such adaptations may influence outcomes [1]. Adaptations of EBIs are difficult or even impossible to avoid when interventions developed in research are implemented in various practice settings [4]. Adaptations are often made to successfully translate new interventions into other settings and ensure their fit with the local context, e.g. the organization and the client population [5]. Identifying processes and types of adaptations of EBIs is important for understanding how to improve the fit between an intervention and the context in which practitioners will use the intervention [6]. Thus, knowledge about adaptation is essential in evaluating the effectiveness of an intervention [7].

Adaptations to EBIs challenge the basic assumption that the core components of an intervention must be implemented with fidelity to achieve its intended effectiveness and expected outcomes [8–10]. Much of the fidelity versus adaptation debate has been theoretical [1], but Durlak [11] has estimated that as much as 80% of an EBI’s prescribed activities may be omitted during implementation. Studies have shown that adaptation to the local context may have both positive and negative impacts on implementation [12–15]. For example, Moore et al. [1] found that adaptations that are made to improve fit do not detract from the core components’ positive influence on clinical outcomes. In general, research examining adaptation outcomes show mixed results [2, 4, 16]. Evidence is lacking regarding which outcomes (intervention or implementation outcomes) are influenced by adaptations and whether certain types of adaptations are more likely to influence certain types of outcomes [4].

In the adaptation literature, a distinction is made between adaptations and modifications [1, 2, 17]. Whereas adaptations are planned or proactive [1, 17], modifications are reactive changes made to interventions in response to unanticipated challenges that arise in a given context [17]. Modifications may be implemented somewhat haphazardly for convenience or to save time [4]. In the current study, adaptation refers to planned or purposeful changes to the content and delivery of the new intervention; it does not apply to other original interventions already tested in controlled settings, which adaptation often refers to in literature on adaptation [1, 2, 17, 18].

Adaptations and modifications are often required because EBIs are developed and designed by researchers, programme developers and education organizations separate from clinical practice or other contexts where the intervention will be implemented [2, 4, 7]. In practice, adaptations and modifications are expected when implementing EBIs in routine practice. This recognition has led to the development of a number of frameworks and taxonomies that aim to structure and describe the characteristics of adaptations and modifications [1, 9, 17, 19–22]. Despite these efforts, it remains difficult to fully capture the complexity of adaptation and modification processes, and empirical studies are needed to deepen our understanding of the change processes that interventions undergo when implemented in routine clinical practice [2, 8].

When developing EBIs, stakeholder engagement is advocated as a means to reduce research waste, increase value [23] and reduce the need for adaptation during implementation for a better intervention-context fit [24]. The assumption is that when stakeholders, who have a direct interest in the process and outcomes of a project, research or policy endeavour [24], are involved in designing the intervention from the outset, the credibility of the outcomes will improve [25–27]. Engaging stakeholders manifests itself through methods such as co-design [28]. Co-design and co-creation are applied and understood inconsistently in different fields (e.g., community psychology, implementation science, human-centered design) and may look different in different settings, e.g. whether designers are needed to facilitate the process and whether tools and probs are needed [29, 30]. In this study, co-design refers to a methodological approach where stakeholders are involved in designing interventions, and where designers work with people who are not trained in design work together in the development of an intervention. It is usually recommended that stakeholders participate throughout the design process [31]. The assumption is that co-designing an intervention will ensure that the intervention will meet stakeholders’ present and future needs [31, 32]. In this study, stakeholder engagement is defined as a process with the overall rationale of making use of engagement methods, such as co-design, to increase the quality of a product or an intervention. There are numerous advantages and disadvantages of stakeholder engagement and co-design [33]. For example, stakeholder engagement is considered to improve the quality of the research by enhancing the credibility of the results [34, 35]. Some practical impacts of co-designing processes are enhanced satisfaction and empowerment for patients and relatives and a greater level of creativity among stakeholders and better relationships between different stakeholders cross sectoral. The broader implications of stakeholder engagement and co-design methods in relation to this study are examined in a different article by Kirk et al. [36]. Therefore, the focus of the present article is adaptations and modifications of a health care intervention.

The co-design literature does not use the concepts of fidelity, adaptation and modifications, but it can be assumed that co-design approaches facilitate a high degree of fidelity and require less adaptations and modifications compared with interventions that are not co-designed with stakeholders. However, there is a knowledge gap on the relationship between co-design and adaptations, if we look at empirical studies in which many focus on co-design [30, 37–40] while others focus on adaptations [6, 8, 9, 14]. The question is not whether adaptations are made, but whether co-designed interventions prevent modifications, understood as reactive changes implemented somewhat haphazardly for convenience or to save time, and how these modifications influence the clinical and implementation outcomes.

The WALK-Cph project [41] was developed based on the assumption that involving stakeholders in a co-design process would both facilitate the fidelity of the intervention to increase the mobility of acutely admitted older medical patients and facilitate its implementation in two hospitals in Denmark. This patient group has limited mobility during hospitalization [42] and a high prevalence of hospital-associated disability [43]. The purpose of this study is to describe and analyse adaptations and modifications that were made to the co-designed WALK-Cph intervention and its implementation plan. The aim was to investigate why the health professionals and health care managers made adaptations and modifications to the co-designed WALK-Cph intervention and to examine if and how these changes influenced the clinical and implementation outcomes.

## Methods

This study focuses on adaptations and modifications made to the in-hospital components after the development of the intervention and in relation to a subsequent fidelity study. This study is designed as an ethnographic field study, which is well-suited to study experiences of individuals or groups as well as the social interactions and contextual changes [44] in which adaptations occur. Both an ethnographic field study using participant observations [45, 46], workshops and semi-structured interviews were performed [47]. The study is reported using the Standard for Reporting Qualitative research checklist [48] and the Standard for Reporting Implementation Studies [49].

### The WALK-Cph intervention

The intervention outcome of the WALK-Cph project was a 45 min increase in upright time per day for older medical patients [41]. We have previously shown that older medical patients spend a median of 22 h per day being inactive during hospitalization in our hospital [50], which is in agreement with a general picture of low in-hospital mobility when summarized as level 1A evidence across different patient populations, health care settings, and countries [42]. Low mobility increases the risk of functional decline, loss of independence and death [51, 52]. The designed intervention was considered “new” because it was conceptually a new intervention based on a newly designed programme theory (see Additional file 1) [53] and was co-designed [31].

The intervention was co-designed by the research team, health professionals, patients and relatives of the patients [31] in an iterative workshop process of three workshops. Each workshop lasted between 3 and 4 h and were held in a meeting room in the hospital. The aim of the co-design sessions was to give the stakeholders the possibility to contribute to designing the intervention. The process was facilitated by the research team who coordinated and assisted group discussions and activities during the workshops. The facilitators did not contribute with ideas but encouraged input from participants in their respective groups. Hereafter, the intervention was initiated, and follow-up workshops were held with healthcare professionals and researchers for an overview of adaptations and modification of the intervention. For further details on the co-design process please see Kirk et al., 2021 [36].

The mobility intervention consisted of six in-hospital components (components 1–6) and two post-discharge components (components 7 and 8): (1) welcome folder, (2) WALK-path, (3) posters with physical exercises placed in the wards, (4) physician-prescribed WALK-plan, (5) independent collection of clothes, (6) independent collection of beverages, (7) after discharge, patients with a rehabilitation plan were contacted by phone by a municipal therapist or (8) after discharge, patients without a rehabilitation plan were contacted by phone by a municipal health professionals (see Additional file 2). The core components of the intervention were considered to be the WALK-path (component 2) and the WALK-plan (component 4) [54]. This study focuses on adaptations and modifications made to the in-hospital components after the initiation of the intervention and during a subsequent fidelity study investigating implementation fidelity.

### Study setting

The study was carried out in Denmark, where the health care system is primarily publicly funded from taxes. The Danish welfare state provides free treatment for primary medical care, hospitals, and home-based care services for all citizens. The WALK-Cph project involved four medical departments at three public hospitals in the Capital Region of Denmark. The four departments encompass six medical specialities: (1) endocrinology; (2) infectious diseases; (3) pulmonary diseases; (4) emergency medicine; (5) gastroenterology; and (6) general medicine. Two of the departments, endocrinology (X) and general medicine (Y), were randomized to the mobility intervention before the co-design process. The two intervention departments are situated in different hospitals and municipalities in Denmark [55].

The two departments are similar in size and staff composition. Department X has 24 beds and 36 staff members consisting of nurses (*n* = 18), certified nursing assistants (*n* = 6) and physicians with responsibility in the department (*n* = 12). Department Y has 25 beds and 37 staff members consisting of nurses (*n* = 18), certified nursing assistants (*n* = 11) and physicians with responsibility in the department (*n* = 8). The therapists are organized differently. In Department X, physiotherapists are called for from a central physio- and occupational therapy department when a patient needs to be attended by a physiotherapist. In Department Y, physiotherapists are part of the multidisciplinary team at the department [55].

### Recruitment of participants to the follow-up workshops

#### The participants were recruited from the two intervention departments

(X and Y) including the department of occupational and physical therapy (Hospital X) and the rehabilitation departments in Municipalities Y. Initially, is was the frontline managers who identified the nurses, nursing assistants, therapists and physicians who had been involved in the design process of the intervention and implementation. These health professionals were selected as they have interest in or experience with designing and implementing an intervention. The follow-up workshops ended up having participation from one physician, three nurses, three nursing assistants, three physiotherapists, three occupational therapists and five frontline managers participated (Table 1).

**Table 1:** Health professionals participating in follow-up workshops

Profession	Number	Years of Experience	Sex
Physiotherapists	3	< 2, > 5, > 5 and > 5	4 females
Physician	1	> 10	1 female
Occupational therapists	3	> 5, < 2 and > 2	3 females
Nurses	3	< 5, > 5 and > 10	3 females
Assistant nurses	3	> 5,> 10 and > 10	3 females
Frontline managers	4	> 5, > 10, > 10 and > 10	1 man and 3 women

### Data collection

The data collected for the adaptation and modification analysis in the present study are based on field notes collected during the fidelity study using participant observations and during two follow-up workshops with health professionals and managers, as well as during two subsequent interviews. The fidelity study was conducted over three periods as part of an observational study (4 weeks in each period between September 2018 and March 2019). The first and last authors of this article (JK and MMP) and four research assistants (BG, NS, BSP and RB) were present in both departments during two sessions of 4 h a day observing the health professionals in their daily practice. Two of the researchers (JK and NS) were trained and had several years of experience using participant observation as a method. The other researchers (MMP, BG, BSP and RB) were trained by initially following JK or NS to observe and learn. Then they performed participant observations where JK or NS participated on the sideline. In addition, the researchers talked together every day, to follow up on situational experiences that the individual researcher needed to discuss e.g. ethical dilemmas. These talks were subsequently written down in the field notes.

The researchers focused on both the fidelity of the intervention and modifications and adaptations to the intervention and the implementation plan. The observation of fidelity was guided by the modified Conceptual Framework for Implementation fidelity developed by Hasson et al. [56], and was described in an observation guide. When fidelity factors from the framework were not met or performed differently than described (modified) this was written down. If required by the situation, the researcher asked what had been observed. Participant observations were made by following the health professionals in the department or by standing in the hallway by the department’s WALK-path. This meant that notes were made both as key words and as full sentences and questions that were raised along the way. After each observation day, the researcher went back to the office and wrote out the field notes, which were placed in a common folder on the research department’s drive. Data saturation was achieved when the field observations did not bring new facets to the observations and initial patterns began to emerge in the material. As part of the fidelity study, a common digital diary was used to record reflexive notes concerning thoughts, feelings and observations about adaptations and modifications. These notes were used to discuss pre-understandings and surprises among the researchers and were used as a method to reflect on biases.

The follow-up workshops (September and December 2019) (Table 2) lasted for 3 h in meeting rooms at the hospitals and were led by JK and MMP; several of the co-authors of this study also participated. At these workshops, initial findings were presented and discussed with the participants. Transcribed material from two audio taped, semi-structured interviews held by JK and MMP with one frontline manager from Department X and one implementation champion from Department Y was included. The interviews each lasted approximately 1 h and they were held in meeting rooms at the hospitals. The interview guide was developed with the background of the preliminary results from the fidelity and workshop studies e.g. one finding from the fidelity study, which was put forward in the interviews, was that the welcome folder (an intervention component) was rarely handed out to patients, and the two managers were asked about their considerations on this matter and what they thought could support the use and implementation of the welcome folder. In total, the data material from workshops and interviews consisted of 256 A4 pages (Table 2).

**Table 2:** Data material

Date and Period	Type of Study	Field Notes	Notes from Semi-structured Interviews
September–November 2018	Fidelity I	61 pages	
January–February 2019	Fidelity II	61 pages	
February–Mar 2019	Fidelity III	65 pages	
September 2019	Follow-up workshop I	33 pages	
December 2019	Follow-up workshop II	21 pages	
January 2019	Interview with frontline manager X		5 pages
March 2019	Interview with implementation champion Y		10 pages
Total		241 pages	15 pages

### Data analysis

The data were analysed using the Framework Method [57]. The texts were read twice by JK and an initial qualitative categorization, coding and analysis was performed based on the Adaptation-Impact Framework (AIF) [58]. The AIF [4] includes frameworks from Stirman et al. [2], Moore et al. [1] and Proctor et al. [59] combined in three domains: (1) adaptation or modification descriptions [2]; (2) possible mediating factors [1]; and (3) effect of the adaptation on implementation outcomes [59]. In 2019, Stirman et al. [17] expanded the framework (FRAME) to include components regarding modifications, which are also included in our analysis. In this study, reactive adaptations are referred to as modifications, in line with Stirman et al. [17]. The points of interest in the frameworks are represented in Table 3.

**Table 3:** Frameworks included in the analysis

Years	Framework	Points of interest
2013	Stirman et al.	Who made the adaptations?
		What was adapted?
		What was the level of the adaptation (e.g. content or context, individual patient or group of patients)?
		What was the nature of the adaptation (e.g. tailoring or adding)?
2019	The FRAME framework form Stirman	Was the adaptation planned?
		What were the goals of the adaptation?
		What were the reasons for the adaptation (e.g. sociopolitical, organizational)?
2013	The Framework of Moore	The fit can be either philosophical (i.e. aligned with the views of the practitioners and the organization) or logistical (i.e. aligned with the context and understood as, e.g. capacity, skills, and knowledge)
		The timing of the adaptation can be either proactive (i.e. planned) or reactive (i.e. haphazardly)
		The valence considers whether the adaptation aligns with the intervention’s goals, i.e. its core components, and can be either positive (i.e. aligned), neutral (i.e. neither aligned with nor deviated from) or negative (i.e. deviated from).
2011	Proctor’s framework	Acceptability, adoption, appropriateness, feasibility, fidelity, implementation cost and sustainability

When combining the three frameworks in the AIF, it becomes possible to systematically assess the influence of adaptations on outcomes. In this study, it is only possible to present data on the impact of the adaptations and modifications on implementation outcomes focusing only on the outcomes of acceptability, fidelity and adoption (Additional file 1). Regarding the clinical outcomes, it is only possible to present the expected effect of the adaptations and modifications (unpublished data).

The transcribed data material were first read twice by JK and then condensed, coded and categorized in a coding scheme constructed with the intervention components and implementation strategies on one axis and adaptation and modifications components from the AIF [58] on the other axis. To secure consistency and rigour in the coding and interpretation of the data material [44], the material was divided into two parts. These two parts were deductively coded by MMP and NS, respectively, based on the AIF. The coded material was compared and discussed by JK, MMP and NS until consensus was reached. Afterwards, discussions were held with the rest of the research team (PN, TTT, TB, OA, RB, BSP, BG) and the results were developed. This iterative process resulted in the findings presented in Table 4. The results of the analysis are presented in the form of general patterns in relation to adaptations and modifications of the intervention components and of the implementation strategies.

**Table 4:** Overview of all adaptations to intervention components

Intervention Component	Department X/Y	Number of Adaptations	Content or Context Adaptation	Descriptions of the Adaptations	When Did the Adaptation Occur?
WALK-plans	X	1	Content	Patients who have not been assigned a WALK-plan are marked with a minus sign on the board in the staff room. This is to ensure that all health professionals are made aware that a decision has been made about WALK-plans for the patient in question	Implementation
	X	2	Content	A rack for WALK-plans is installed	Pre-implementation
	X	3	Content	Changes in time and timing of board meetings, where WALK-plans are discussed	Implementation
	X	4	Content	Changes in the interprofessional collaboration in relation to WALK-plans by which the physicians did not have a part in the intervention	Implementation
	X	5	Content	It is discussed whether WALK-plans should be described in the care plan or in the discharge report/rehabilitation plan, and it is agreed that the WALK-plans should be described in the discharge report/rehabilitation plan	Implementation
	X	6	Content	Changing signature on the WALK-plans, so the nurses and physiotherapists become responsible for signing the WALK-plans	Implementation
	X	7	Content	Changes in the responsibility for handing out WALK-plans with shifts from physicians to nurses and physiotherapists	Implementation
	X	8	Content	WALK-plans (home) are not handed out to patients	Implementation
	X	9	Content	In-hospital WALK-plans and WALK-plans (home) are merged into one WALK-plan	Implementation
	X	10	Content	Sticker with WALK-Cph logo is attached to envelope with welcome folder	Implementation
	Y	11	Content	Patients who have not been assigned a WALK-plan are marked with a minus sign on the board in the staff room. This is to ensure that all health professionals are made aware that a decision has been made about WALK-plans for the patient in question	Implementation
	Y	12	Content	The WALK-plans are placed behind the patient’s bed, so it becomes visible for both patients and health professionals	Implementation
	Y	13	Content	The establishment of a whiteboard with clips and coloured magnets for WALK-plans located in the staff’s room. This is done to create greater visibility and systematization of the WALK-plan practice	Implementation
	Y	14	Content	Changes in the responsibility for signing and handing out WALK-plans shifting from physicians to physiotherapists	Implementation
	X	1	Context	Implementation champions become responsible for board meetings when frontline managers are unavailable	Implementation
	X	2	Context	The WALK-plans are placed behind the patient’s bed, so it becomes visible for both patients and health professionals	Implementation
	X	3	Context	WALK-plans are expanded in the rehabilitation department so all patients, and not only patients enrolled in the WALK-trial, receive a WALK-plan	Implementation
WALK-path	X	1	Content	A sign indicating the length of the WALK-path in metres is placed on the wall in the department	Implementation
	X	2	Content	Posters are placed on the walls in the patient rooms	Implementation
	X	1	Context	The WALK-path is moved into the new building when the department moves	Scale up
	X	2	Context	Two therapists help mobilize patients at lunch time	Implementation
	X	1	Content	The green chairs disappear	Implementation
	Y	1	Content	The blue chairs disappear. The chairs were similar to the other chairs in the ward and were not marked as WALK-chairs	Implementation
	Y	2	Content	Marking of blue chairs to ensure that they do not disappear from their location by the posters	Implementation
	X	1	Content	Small whiteboards are used periodically by health professionals and patients	Implementation
	X	2	Content	The small whiteboards are brought back to life; the physiotherapists start counting the daily number of rounds that patients have walked	Implementation
Independent collection of clothes	X	1	Content	Signs with sizes and descriptions of clothes are placed on the shelves in the closet. The placement of clothes in the closet is changed, so smaller garments are placed at the bottom of the closet so that patients of smaller stature can reach their sizes	Implementation
	X	2	Content	Stickers are placed on the outside of the closets to indicate contents	Implementation
Welcome folder	X	1	Content	Information about the project is not given when handing out welcome material and therefore a WALK-logo is added as a reminder	Implementation
Independent collection of beverages	X	1	Content	The refrigerator is moved due to fire hazard. Patients can no longer access the refrigerator and collect beverages	Implementation
	Y	1	Content	No one is responsible for ensuring that fresh coffee and other beverages are placed on a table in the ward for self-service	Pre-implementation
Others	X	1	Content	Reward in the form of cake for staff and patients when the patients in the department had walked 10 km in total	Implementation
	X	2	Content	The board of directors are informed about the project and how many kilometres patients have walked in total	Implementation
	Y	1	Content	Magnets are placed on the overview board to indicate which patients have a WALK-plan	Implementation
	Y	2	Content	The whiteboards to mark rounds are abandoned due to formal rules for this hospital	Pre-implementation
	Y	3	Content	Posters with research findings are placed on the wall along the WALK-path to motivate health professionals	Pre-implementation
	Y	4	Content	Posters with research findings are removed, so that they are no longer visible	Implementation
	Y	1	Context	Due to an organizational change in the Danish health care system, where smaller hospitals are merged with larger ones, Department Y closes	Implementation
Implementation strategies	X	1	Content	Written material placed by the computers as reminders are forgotten	Pre-implementation
	X	2	Content	The physicians talk about the project at physicians’ conferences and at board meetings	Implementation
	X	3	Content	Increased communication efforts (at staff and morning meetings) about the WALK-intervention to spread the message about WALK	Implementation
	X	4	Content	The operational target board is not used in relation to Walk-plans; no operational target has been set for Walk-plans	Implementation
	X	5	Content	The frontline manager nurse receives coaching in relation to the management of the project	Implementation
	Y	1	Content	Increased communication efforts about the WALK-intervention in the group of nursing staff to spread the message of WALK	Implementation

. First, adaptations and modifications were described using the framework from Stirman et al. [2, 17] to see how adaptations or modifications influenced outcomes. Then, the framework from Proctor et al. [59] was used to describe which types of adaptations or modifications influenced which outcomes and in which directions. Finally, constructs from the Moore et al. framework [1] were used to explain why and how outcomes were influenced [4] (Tables 5, 6, 7, 8, 9, 10, 11 and 12). A second deductive analysis was done using the AIF framework retrospectively and is presented with three empirical examples at a micro, meso and macro level.

**Table 5:** Content-based adaptations in Department X and Y [2, 15]

Intervention Components	Department	WHO Made the Modifications and Adaptations?						At what level?			
		Individual Practitioners	Team of Practitioners^**a**^	Non-programme Staff	Patients and Relatives	Authorities	Unknown	Individual Practitioners	Group Level^**b**^	Department Level	Hospital Level
WALK-plans	X	●	●							●	
	Y	●	●	●						●	
WALK-path	X	●	●	●					●	●	
WALK-chairs placed at rest areas	X			●	●				●		
	Y	●		●	●				●		
Small whiteboards are placed on the wall next to the WALK-path	X		●						●	●	
Independent collection of clothes	X		●						●		
Welcome folder	X	●								●	
Independent collection of beverages	X					●					●
	Y	●								●	
Others	X	●	●						●	●	
	Y	●	●			●			●		●
Implementation strategies	X	●						●		●	
	Y	●								●	

^a^Team of practitioners are implementation champions and managers

^b^Group level are group of patients

**Table 6:** Content-based adaptations and modifications in Department X and Y [2, 15]

Intervention Components	Department	The Nature of the Adaptation and Modifications?						The Intent of the Adaptation and Modifications?					
		Adding	Removing	Refining	Tailoring	Expanding	Delaying	Reach	Feasibility	Satisfaction	Engagement	Security	Cost Reduction
WALK-plans	X	●	●	●	●	●		●	●	●	●		
	Y	●			●			●	●	●	●		●
WALK-path	X	●			●		●		●		●		
WALK-chairs placed at rest areas	X		●						●				
	Y	●	●						●				
Small whiteboards are placed on the wall next to the WALK-path	X	●						●					
Independent collection of clothes	X	●						●					
Welcome folder	X	●							●				
Independent collection of beverages	X		●									●	
	Y						●		●				
Others	X	●	●					●			●		
	Y	●	●					●			●		●
Implementation strategies	X	●	●					●	●		●		
	Y	●									●

**Table 7:** Context-based adaptations and modifications in Department X and Y [2, 15]

Intervention components	Department	Who Made the Adaptations?						Context Adaptation Level?			
		Individual Practitioners	Team of Practitioners	Non-programme Staff	Patients and Relatives	Authorities	Unknown	Format	Setting	Personnel	Population
WALK-plans	X	●	●					●	●	●	
WALK-path	X		●	●				●		●	
WALK-chairs placed at rest areas	X			●	●					●	
Small whiteboards are placed on the wall next to the WALK-path											
Independent collection of clothes											
Welcome folder											
Independent collection of beverages											
Others	Y					●			●		
Implementation strategies

**Table 8:** Context-based adaptations and modifications in Department X and Y [2, 15]

Intervention Components	Department	The Nature of the Adaptation?						The Intended Goal?					
		Adding	Removing	Refining	Tailoring	Expanding	Delay	Reach	Feasibility	Satisfaction	Engagement	Security	Cost Reduction
WALK-plans	X	●				●		●			●		
WALK-path	X	●			●				●		●		
WALK-chairs placed at rest areas	X												
Small whiteboards are placed on the wall next to the WALK-path													
Independent collection of clothes													
Welcome folder													
Independent collection of beverages													
Others	Y		●										●
Implementation strategies

**Table 9:** Content-based adaptations in Department X and Y [1]

Intervention Components	Department	Did the Adaptation or Modification Detract from Core Components?		Was It an Adaptation or a Modification?		The Rationale for the Adaptation/Modification?	
		Yes	No	Planned	Haphazard	Philosophical Fit	Logistical Fit
WALK-plans	X	●	●	●	●	●	●
	Y	●	●	●	●	●	●
WALK-path	X	●	●		●		●
WALK-chairs placed at rest areas	X		●		●		●
	Y		●		●		●
Small whiteboards are placed on the wall next to the WALK-path	X	●		●	●	●	
Independent collection of clothes	X		●	●	●		●
Welcome folder	X		●	●			●
Independent collection of beverages	X	●			●		●
	Y	●			●		●
Others	X		●	●		●	
	Y	●	●	●	●	●	●
Implementation strategies	X		●	●	●	●	●
	Y	●		●			●

**Table 10:** Context-based adaptations and modifications in Department X and Y [1]

Intervention Components	Department	Did the Adaptation or Modification Detract from Core Components?		Was It an Adaptation or a Modification?		The Rationale for the Adaptation/Modification?	
		Yes	No	Planned	Haphazard	Philosophical Fit	Logistical Fit
WALK-plans	X		●	●	●	●	●
WALK-path	X		●		●		●
WALK-chairs placed at rest areas							
Small whiteboards are placed on the wall next to the WALK-path							
Independent collection of clothes							
Welcome folder							
Independent collection of beverages							
Others							
	Y	●		●		●	
Implementation strategies

**Table 11:** Content-based adaptation and modifications in Department X and Y [43]

Intervention Components	Department	Was Change Made to the Intervention or the Implementation?		Intervention and Implementation Outcome Reach (Effect Positive or Negative)						
		Intervention	Implementation	Acceptability	Fidelity	Adoption	Positive Effect (Implementation Outcome)	Negative Effect (Implementation Outcome)	Positive Effect (Clinical Outcome)	Negative Effect (Clinical Outcome)
WALK-plans	X	●	●	●	●	●	●	●	●	●
	Y	●	●	●	●		●		●	●
WALK-path	X	●			●			●	●	●
WALK-chairs placed at rest areas	X	●			●			●		
	Y	●			●			●	●	
Small whiteboards are placed on the wall next to the WALK-path	X	●			●		●	●	●	●
Independent collection of clothes	X	●			●	●	●			
Welcome folder	X		●		●		●			
Independent collection of beverages	X	●			●			●		
	Y	●			●			●		
Others	X	●	●	●		●	●		●	
	Y	●	●	●	●		●	●	●	●
Implementation strategies	X		●	●	●	●	●	●	●	
	Y		●		●		●		●

**Table 12:** Context-based adaptation and modifications in department X and Y [43]

Intervention Components	Department	Was Change Made to the Intervention or the Implementation?		Intervention and Implementation Outcome Reach (Effect Positive or Negative)						
		Intervention	Implementation	Acceptability	Fidelity	Adoption	Positive Effect (Implementation Outcome)	Negative Effect (Implementation Outcome)	Positive Effect (Clinical Outcome)	Negative Effect (Clinical Outcome)
WALK-plans	X	●	●		●	●	●		●	
WALK-path	X	●			●			●	●	●
WALK-chairs placed at rest areas										
Small whiteboards are placed on the wall next to the WALK-path										
Independent collection of clothes										
Welcome folder										
Independent collection of beverages										
Others	Y		●		●		●		●	
Implementation strategies										

### Trustworthiness

The trustworthiness of the findings is sought to be obtained through credibility by of the use of methods such as participant observations and workshops, which are well established in qualitative research and are well-suited to study adaptations and modifications in daily practice [60]. Credibility was further obtained by the researchers’ engagement in the field with participants both by participant observations over three periods and by several workshops, where the researchers invested time to become familiar with the context, to build trust with the participants and to obtain rich data [60]. In the workshops, initial findings were presented to the participants to continue the analysis and to validate concepts as modifications and adaptations to further strengthen the credibility [61]. The feed back of the initial findings gave the participants the opportunity to comment on the findings and thereby continue the analysis on a more abstract level. Transferability of the finings is difficult to achieve as the modifications and adaptation are tied to the times and the contexts in which they are found. Through the study, thick descriptions of the context has been attempted, e.g. in the retrospective analysis, to provide a possibility to transfer the findings to other contexts [62]. Trustworthiness was also strengthened by using method triangulation in terms of using data both from the field study, the workshops and the interviews [62]. Finally, reflexivity was sought by means of the researchers’ use of a common digital diary as a method to reflect on biases and secure confirmability [60].

## Results

### Numbers of adaptations and modifications

Many different types of adaptations and modifications were made throughout the implementation of the WALK-Cph intervention (see Table 4). In total, 21 adaptations (planned) and 23 modifications (haphazard) were made. Of the 44 modifications and adaptations, 33 were made in Department X and 11 in Department Y. Thirty-eight of the modifications and adaptations were made in the implementation phase, five modifications and adaptations were made in the pre-implementation phase and only one adaptation was considered for the scale-up phase. Thirty-seven of the 44 adaptations and modifications were made regarding content and seven were made regarding context.

### Content adaptations and modifications

The adaptations and modifications were mostly initiated and executed by individual practitioners (frontline managers) and teams of practitioners (implementation champions and managers). The adaptations and modifications to the content were predominantly made at a group level (for older medical patients) and at a department level (for health professionals). The only adaptation made at the hospital level was that white boards were not put up on the walls due to formal rules in one of the hospitals, which was located in a protected historic building. One modification was made at a hospital level: all refrigerators in the hospital were removed from the department so that they were no longer accessible to patients. This was a result of a fire in a refrigerator in another department, which meant that patients could no longer collect beverages themselves, with a negative impact on intervention fidelity.

Most of the content adaptations and modifications related to adding or removing elements, e.g. adding a rack for WALK-plans to make them visible to all health professionals. The intended goals of the content modifications and adaptations were divided between increasing reach, engagement of the intervention and implementation and feasibility. There was no clear pattern on whether content adaptations and modifications detracted from core components. Also, the rationale was mostly for a logistical fit, e.g. stickers were placed on the common-area closets to guide the patients in choosing correct sizes.

### Context adaptations and modifications

Context adaptations and modifications were characterized by being initiated and executed by individual practitioners (frontline managers), teams of practitioners (implementation champions and managers), patients and relatives, non-programme staff and authorities. There was no clear pattern on the levels at which context adaptations and modifications were made. They occurred at levels of format (e.g. the intervention), setting (the intervention was delivered in a different setting) and personnel (the intervention was delivered by changing personnel). Context adaptations and modifications consisted of adding elements with an intended goal of engagement. No context adaptations and modifications detracted from core components and were predominantly made to improve logistical fit.

Most of the content and context adaptations and modifications made on the intervention had a mixed result regarding an intended fidelity effect, e.g. a change in timing of board meetings may have resulted in non-attendance of physiotherapists and thus of discussion of WALK-plans; and a rack for WALK-plans may have facilitated discussion of WALK-plans. However, the expected effect on the clinical outcome of the adaptations and modifications was predominantly positive.

### Retrospective modifications analysis

The results from the retrospective analysis of the complexity of the modifications made haphazardly to the WALK-Cph intervention and implementation are presented through three examples: (1) chairs that went missing (micro level), (2) physicians’ lack of commitment to the intervention (meso level) and (3) closure of the intervention in Department Y (macro level).

### The missing chairs

As part of the intervention, two WALK-chairs were placed by the posters showing the exercises in both departments. Throughout the co-design process, these WALK-chairs became a controversial topic. The implementation champions and the managers wanted the chairs to be ergonomically designed with armrests so that older patients could rise correctly from the chairs. The managers explained:” Our current chairs are not usable as they are too heavy, and many do not have armrests” (manager, Department X, interviews). It was also important that they were not too heavy so that the patients could place them correctly when using them for the exercises illustrated on the posters. The WALK-chairs in Department X were to be placed in a rest area with other chairs that were too heavy to move. Therefore, the WALK-chairs stood out by having a different colour. The WALK-path was green, and it was requested that the WALK-chairs had a similar green colour to illustrate that they were part of the intervention. In Department Y, the WALK-chairs could not differ from the other chairs in colour and shape due to local regulations. All chairs in the department were ergonomically correct (blue chairs with armrests). Thus, the colour of the chairs corresponded to the colour of the WALK-path. The implementation champions put stickers on the two WALK-chairs to make them stand out. In both departments, the hospital architects had to deliver the WALK-chairs. This was not a challenge in Department Y, because it was a standard chair. In Department X, however, the colour was a challenge. The architect expressed:” There are rules in the region for which colours may be used in the departments” (architect, Department X, field notes). Green was not in the standard range for furniture at the hospital. Therefore, the architect ended up buying paint and painting the WALK-chairs himself before they could be delivered to the department.

The observations showed that in both departments, the WALK-chairs disappeared from their designated areas. Many times, the researchers noted in their observation guide: “Today there is only one green chair placed in the rest area” (researchers, field notes). The WALK-chairs were not considered a core component of the intervention, so the lack of WALK-chairs does not impair the core components of the WALK-intervention. The implementation champions and the managers generally did not know where the WALK-chairs had gone. A manager expresses:

“Every day I look for the green chairs and put them back where they belong but the next day they are gone again. That is thought-provoking” (manager, Department X, interviews)

The observations showed that patients and relatives borrowed the WALK-chairs and took them into the patient rooms if a chair was missing or if there were not enough chairs in the room. The green WALK-chairs were easier to move and conspicuous for patients and relatives due to their colour, which may be why they were moved. The same was evident for staff in the neighbouring department (non-programme staff). The modifications were made haphazardly with a logistic fit that had a negative effect on the fidelity of the WALK-intervention because the patients were not able to use the WALK-chairs to exercise as illustrated on the posters.

### Physicians’ lack of commitment to the implementation of the intervention

The physicians were considered to have a very central role in the intervention to achieve the desired clinical outcome. They had to prescribe WALK-plans for all patients capable of walking and encourage all patients to be physically active during hospitalization. An implementation champion describes: “The physicians are very central to the implementation of the intervention as their words are full of authority for many patients” (implementation champions, Department Y, interviews). They also had to participate in daily board meetings to discuss which patients were eligible for a WALK-plan. In the co-design process, all stakeholders were convinced that the intervention was acknowledged on a philosophical level by physicians, therapists and nursing staff. The physicians’ participation was expected to be a core component in relation to the WALK-plans because all stakeholders were confident that the patients would be more compliant and follow physicians’ recommendations and would be physically active if a physician told them to be. An implementation champion stated:

“If the physicians, who already prescribe the needed medicine, also - just call it - prescribe uptime. I think that will mean something to most patients. The older generation is still very loyal to authority” (implementation champions, Department Y, workshops).

The observations showed that in practice, the physicians in both departments did not carry out their part of the intervention for many different reasons. When asking one of the implementation champions about this observation, she answered:

We feel that they [the physicians] have not been interested in being involved in any of it, that it is quite clear. They have not really engaged themselves into the project. (Implementation champions, Department Y, workshops)

The only physician who attended the workshops acknowledged this and said:

“You are absolutely right. However, there are many different reasons why my colleagues have not shown so much interest” (physicians, Department X, workshops)

Based on these experiences, the research group decided to systematically examine the physicians’ perspective and experiences with the intervention. The results showed multiple reasons for the physicians’ lack of commitment. Firstly, the physicians found the interior of the departments unfit for the intervention [63]. Secondly, the physicians did not see mobility as part of their job and responsibility. Finally, the physicians expressed lack of time and resources and therefore unwillingness to accept additional workload [63]. The consequence of the physicians’ lack of commitment in the implementation of the intervention was that the implementation champions and managers made multiple modifications to the intervention to enable the physicians’ to participate. I.e. on some days, the board meeting was changed to fit the schedule of the physicians and on other days the board meeting was cancelled if the physicians did not have time to participate. The manager commented 1 day on these modifications on a board meeting:” I spend a lot of time finding the physicians and trying to persuade them to attend the board meetings” (manager, Department X, field notes). Also, the nurses and the physiotherapists began to sign the WALK-plans instead of the physicians. Despite all these attempts to accommodate the physicians, the intervention was modified so that only the nurses and physiotherapists were responsible for the intervention. The modifications were made haphazardly with both a philosophical and a logistic fit that had a negative effect on the fidelity and adoption of the implementation of the WALK-intervention and with an excepted negative effect on the clinical outcome.

### Closure of intervention department Y

In the implementation phase, Department Y closed as a result of political decisions. An implementation champion expresses:

“We are very sad that the department has to close. We have adapted the intervention to suit most of the patients in the department” (implementation champion, Department Y, interviews).

Despite the fact that the intervention departments were selected through randomization, and the intervention and the implementation were developed through a long co-design process, it was external factor that became the reason why the intervention and implementation were modified many times in Department Y. Due to the closure of the department, more therapists was relocated to other departments and therefore there were fewer therapists to hand out WALK-plans. When asking an implementation champion about this, she answered:

Department Y is in bad shape right now; that is, because a lot of health professionals have stopped, and the beds are closed. My colleagues have many other things on their minds than remembering that we must hand out WALK-plans. (Implementation champion, Department Y, interviews)

Every day, the few therapists in the department tried to hand out WALK-plans. However, it was done more randomly than described in the intervention, because they also had other tasks to perform. The managers resigned their jobs and therefore there was no managerial support for the project throughout the implementation phase. The consequence was that project was given less attention. The modifications were made haphazardly with a logistic fit that had a negative effect on both the fidelity and adoption of the implementation and a negative effect on the clinical outcome.

## Discussion

The aim of the study was to investigate why health professionals and health care managers made adaptations and modifications to the co-designed WALK-Cph intervention and to examine if and how these changes influenced the clinical and implementation outcomes. The findings showed that 44 modifications and adaptations were made throughout the implementation of the WALK-Cph intervention. Most of the adaptations and modifications were made regarding the contents of the intervention.

The findings from the retrospective modifications’ analysis show that modifications were ongoing and both situationally and contextually shaped. The findings illustrate how the implementation of the WALK-Cph intervention occurred on different contextual levels in a social field between people and the daily practice in which they were involved. Despite a large and extensive co-design effort to develop the intervention and an implementation plan that was targeted to the population and the context, there were still many adaptations and haphazard modifications along the way that were not predicted or accommodated before the implementation of the WALK-Cph intervention was initiated.

Our findings showed that implementation champions and managers made both planned adaptations and haphazard modifications to better fit the content and context. This finding is in line with previous studies, which have reported both adaptations and modifications to EBIs [8, 9, 64–66].

Most of the adaptations and modifications were made on the content in the form of adding or removing elements and tailoring other elements. This predominance of content changes is consistent with previous studies [2, 13, 66]. Contextual adaptations and modifications were less common than content changes in our study. This result is in contrast to Stirman et al. [2] who found context adaptations and modifications to be the next most frequent type of change. Kemp et al. [67] have emphasized context as a potential precondition, moderator or mediator of implementation processes and outcomes. The low number of context adaptations and modifications found in our study may be because the intervention was developed in an extended co-design process with stakeholders, which could have resulted in a better context fit of the intervention from the outset of the implementation [31]. The fit may also have benefitted from the in-depth cultural analysis of the intervention departments involved, which was carried out by the research team. This analysis identified key determinants of mobility of older medical patients during hospitalization [55], knowledge which was also included as part of the co-design process.

Another noteworthy result was the difference in the number of adaptations and modifications made between the two intervention departments. Department X made three times as many adaptations and modifications as Department Y, despite the fact that the departments are similar in many ways, e.g. in size and staff composition. For improved understanding of adaptations and modifications, Escoffery el al [64]. requested descriptions of critical changes and the consequence of these. One example of important adaptations and modifications that emerged was the physicians’ lack of commitment to the implementation of the intervention, which resulted in multiple modifications to comply with the physicians’ daily practice. This affected the interprofessional collaboration, and in Department Y, the physiotherapists quickly took over the general responsibility for the project and for handing out WALK-plans. This was due to a philosophical fit because mobility is a core competence for therapists, something that was reflected in few context adaptations and modifications as the intervention was adapted to the cultural practice of therapists [55, 68]. These adaptations meant that part of the programme theory (the assumption that the intervention should be interprofessional for most favourable results) was not fulfilled. In contrast, in Department X, the managers and implementation champions struggled to get the physicians involved in the project for a long time. This resulted in Department X making many more adaptations and modifications than Department Y. Despite these attempts, only a few physicians ended up being involved in the project.

Despite the fact that stakeholders were involved in co-designing the intervention and its implementation, as many as 44 adaptations and modifications were made. This was a somewhat surprising finding because the literature suggests that involving stakeholders in co-design processes is an approach to improve the fidelity of interventions. Pérez et al. [69] described how many studies have an implicit assumption: the more an intervention is adapted, the more likely it is that fidelity is threatened with the consequence of reduced effectiveness. This perspective suggests that the intervention is an invariant and the users are passive subjects who always implement the intervention with fidelity. In contrast to this view of adaptation/fidelity, our results showed that both adaptations and modifications can be considered cognitive processes involving managers and implementation champions struggling to give meaning to the intervention during its implementation [70] even though they themselves developed the intervention and the implementation plan. An organization such as health care is not a static setting but is constantly evolving and dynamic. Thus, it is inevitable that unforeseen changes will occur that are likely to affect the intervention and its implementation [71] regardless of whether a co-design process was used as recommended [69]. Most frameworks used in implementation science emphasize an active view of organizational context, recognizing that the organization in which implementation occurs is not merely a passive backdrop to implementation [72].

Some adaptations were systematically planned, whereas others occurred more haphazardly. According to descriptions by Moore et al. [1], Stirman et al. [2, 17] and Kirk et al. [73], both adaptations and modifications are always conscious and explicitly recognized changes. However, the question is whether adaptations can also occur more implicitly, without much conscious awareness. The notion of behaviours being guided by non-conscious cognitive processes is not novel. This seems likely in light of research that demonstrates that many behaviours are at least semi-automatic responses to cues triggered by associations outside of our conscious awareness or control [74]. Interest in dual processing approaches has increased significantly, with much attention focused on behaviours resulting from non-conscious processing. Dual process approaches posit that our behaviours are the result of two cognitive processes operating in parallel: an instinctive process and a deliberate, reflective process [75]. Dual process approaches specify a number of boundary conditions that moderate the relationship between conscious and/or non-conscious processes, including cognitive load, stress, and physical or emotional exhaustion [74]. There is strong evidence suggesting that conscious processing decreases steadily over a normal working day as cognitive resources become depleted [76]. We have not found any research on whether or the extent to which adaptations and modifications are the result of non-conscious processes. Such unconscious behaviours challenge existing adaptation frameworks, which have been developed to help control and standardize adaptation processes (e.g. 11 steps in adaptation processes [9]) by potentially functioning completely differently than usually assumed.

The results from this study show that implementing EBIs is a highly dynamic and adaptive process in which the EBIs are adapted to the organization in which the implementation occurs and the organization is adapted to the intervention [77]. The three examples of haphazard modifications in this study show that modifications are ongoing and situationally and contextually shaped. The findings underscore the contextual nature of implementing EBIs as occurs in a social field among people and as part of different contextual levels. Although implementation science originated from the evidence-based medicine movement [78], which views highly controlled interventions as the gold standard, implementation science must be considered a social science where social relations, the context and interdependencies between many factors play a crucial role in influencing the results. The complexities of implementation processes distance implementation science from its medical roots.

The results from this study show that although users are involved in the design of EBIs, it is impossible to predict and account for all eventualities that might occur in the future. In relation to the design of EBIs, it therefore becomes relevant to ask how to best facilitate making EBIs flexible to allow them to be adapted and modified in relation to various contexts and populations and still retain their effectiveness. How can flexibility be built into interventions to optimize their effectiveness in different contexts? This is an area of enquiry that warrants further studies for improved understanding of how EBIs can have the most impact on important outcomes.

### Limitations and strengths

The findings of this study are difficult to generalize to other countries, settings or EBIs. Different adaptations and modifications may be relevant with other EBIs. Although this study makes use of the frameworks of Moore et al. [1], Stirman et al. [2, 17] and Proctor et al. [59] and Kirk [58], the results raise the issue of whether these frameworks can accommodate the social context and the complexity embedded in the context. When interviews are used as the only method to obtain data on adaptations and modifications, it can be especially difficult for health professionals to explicitly recognize and report these. Another limitation is the lack of use of organizational-level profiles of the two departments [65]. However, we have tried to show how adaptation occurs empirically at different levels. Organizational-level profiles early in the co-design phase could perhaps have contributed with additional contextual knowledge about the two departments’ approach to adapting initiatives in general.

A further limitation of the study is the lack of involvement of the patients and of knowledge regarding which adaptations and modifications they made while in carrying out the intervention. In the field observations, some patient-introduced adaptations and modifications were studied but this was not done systematically. Instead, a systematic interview study was conducted with the patients where their opinions, attitudes, and experience with the intervention was explored [79].

Finally, it may be discussed whether the use of only three outcomes from Proctor’s framework had an impact on the results. Acceptability, fidelity and adoption were chosen in this study as these outcomes are highly relevant in the earlier stages of implementation. Proctor et al. [59] also suggest feasibility as a relevant outcome in the early implementation phase. However, using feasibility as an outcome would have required that the study was designed in a more iterative prototyping approach where we develop while testing.

A strength of this study was the use of ethnography with participant observations, where the participants are followed in their daily practice [80]. This method is suitable for revealing the complexity and social reality when interventions are adapted and adopted. Ethnographic research explores what the health professionals say and do, and their relationship with others. Thereby, ethnography is useful for understanding the collective and non-rational dimensions of organizations [68]. By being present in the daily practice of the health professionals, it became clear that parts of the intervention were modified unconsciously e.g., nurses continued to serve patients who had a walking plan, without even noticed it as it was a part of normal practice. Thereby ethnography and participant observations became inherently contextual which shape the interplay between the intervention and the context in which it is implemented.

Finally, there was little researcher involvement in adapting the intervention, allowing us to assess adaptation and modification in naturalistic daily clinical practice and to test the AIF model, which had not been empirically tested previously [58].

## Conclusions

Although an extensive co-design process was carried out to facilitate fidelity of the WALK-Cph intervention, this study showed that many adaptations and modifications were still made to both the intervention and its implementation plan. On the one hand, this could indicate that the co-design process had a small effect. On the other hand, it could indicate that adaptations and modifications are ongoing and both situationally and contextually shaped, which can challenge the assumption and the desire to be able to plan and control changes. The results raise questions of whether all changes are consciously made and point to the need for research to understand how flexibility can be built into EBIs.

This study is an important empirical and analytical contribution to the implementation science field, especially in relation to the fidelity-adaptation debate. It contributes with a perspective on adaptations and modifications as non-conscious processes, which could challenge the established framework developed in the field.

## Supplementary Information

**Additional file 1.**

**Additional file 2.**

## Data Availability

The datasets used and analysed during the current study are available from the corresponding author on reasonable request.

## Abbreviations

EBI: Evidence-based intervention
WALK-Cph: WALK-Copenhagen
