# Supplementary material for: Adaptations and modifications to a co-designed intervention and its clinical implementation: a qualitative study in Denmark
Source: BMC Health Serv Res. 2021 Oct 16;21:1108. doi: 10.1186/s12913-021-07142-4 (PMC8520628; doi:10.1186/s12913-021-07142-4)
Supplement: Supplementary file 1 — Additional file 1. [file 12913_2021_7142_MOESM1_ESM.docx]

**Appendix 1: PROGRAM THEORY OF INTERVENTION AND IMPLEMENTATION STRATEGIES**

A: INTERVENTION

| **STRATEGY** | **IF…** | **THEN…** | **INTENDED OUTPUTS** | **INTENDED OUTCOMES** |
| --- | --- | --- | --- | --- |
| WALK-PATH | the health professionals motivate and refer the patients to the WALK-path on a daily basis | the patients will gain knowledge and understanding of the importance of being active during hospitalisation | If the patients use the WALK-path on a daily basis | he patients will increase their daily number of steps during hospitalisation |
| WELCOME BOOKLETS FOR PATIENTS | the health professionals provide the patients with welcome booklets upon hospitalisation and express the importance of activity during hospitalisation | the patients will gain knowledge and understanding of the importance of activity during hospitalisation and will change behaviour | If the patients hear and understand the message of activity and read the booklet | The patients will be more active during hospitalisation and after discharge and will thereby increase their daily number of steps |
| POSTERS WITH DIFFERENT EXERCISES IN THE ROOMS | the health professionals motivate the patients to do the exercises on the posters | the patients will be reminded of the importance of activity and training and be inspired by seeing how easily it can be done | If the patients do the exercises described on the posters | The patients will reduce their risk of decreased level of functioning and sustain/increase their muscle strength, which will support their possibilities to remain active |
| PRESCRIPTION OF WALK-PLAN | the medical doctor prescribes, signs and hands out WALK-plans to the patients on a daily basis | the doctor’s authority will help the patients understand the importance of activity and that activity is part of the treatment | If the patients abide to the prescription of activity through the WALK-plan | The patients will increase their number of steps during hospitalisation and after discharge |
| UNAIDED PICKING UP OF CLOTHES | the health professionals motivate and refer the patients to unaidedly pick up clothes from the closets | the patients will gain knowledge and understanding of the importance of being mobile during hospitalization and thus of the staff not serving them | If the patients walk to the closets | The patients will increase their daily number of steps during hospitalisation |
| UNAIDED PICKING UP OF BEVERAGES | the health professionals motivate and refer the patients to unaidedly pick up beverages from the beverage cart | the patients will gain knowledge and understanding of the importance of being mobile during hospitalization and thus of the staff not serving them | If the patients pick up beverages from the beverage cart | The patients will increase their daily number of steps during hospitalisation |
| **THE REHABILITATION UNIT** |  |  |  |  |
| FOLLOW-UP FOR PATIENTS WHO HAVE A REHABILITATION PLAN | the physiotherapists call the patients within the first week after hospital discharge and ask about the patients’ use of the WALK-plan | the patients’ respect for the physiotherapists’ competence will lead to the patients’ understanding the importance of activity when they have returned home | If the patients abide by the activity through the WALK-plan | The patients will increase their number of steps after being discharged |
| **HOME CARE** |  |  |  |  |
| FOLLOW-UP THROUGH NURSING ASSISTANT | the nursing assistant follows up on the residents’ WALK-plans when visiting them at home | the residents’ trust in their home care will lead to the residents understanding the importance of activity when they have returned home | If the residents abide by the prescribed activity through the WALK-plan at home | The residents will increase their number of steps after being discharged |

**B: IMPLEMENTATION STRATEGIES**

| **STRATEGY** | **IF…** | **THEN…** | **INTENDED OUTPUTS** | **INTENDED OUTCOMES** |
| --- | --- | --- | --- | --- |
| TAKE LEADERSHIP | the leaders tell about and ask about the walk project on staff meetings, therapist group meetings and in daily life | the therapists will gain knowledge about the project and learn that the project is prioritised by the management | Management prioritising the WALK-Cph project will encourage the therapists to perform tasks related to WALK-Cph | The possibility for the therapists to acknowledge the project (acceptability) and implement the project with fidelity will increase |
| FOLLOW-UP EDUCTATION AND TRAINING OF NEW THERAPISTS | those responsible for the implementation educate and train the newly hired therapists about WALK-Cph | Newly hired therapists will gain knowledge and skills about the WALK-Cph project and its tasks | If the therapists understand and acknowledge the purpose of WALK-Cph through education and training, they will perform the tasks related to the project | Acceptability, adoption and the possibility of implementing the project with fidelity increases |
| CHANGING THE PHYSICAL SURROUNDINGS | If the people responsible for the implementation change the physical surroundings (more walkers, posters, green resting chairs, small whiteboards) | These materials will remind the health professionals (nudging) of the importance of motivating the patients to increased activity during hospitalisation | If the physical surroundings change, it will lead to changed behaviour from the health professionals | The possibility of the project being implemented with fidelity will increase |
