# Supplementary material for: Adaptations and modifications to a co-designed intervention and its clinical implementation: a qualitative study in Denmark
Source: BMC Health Serv Res. 2021 Oct 16;21:1108. doi: 10.1186/s12913-021-07142-4 (PMC8520628; doi:10.1186/s12913-021-07142-4)
Supplement: Supplementary file 2 — Additional file 2. [file 12913_2021_7142_MOESM2_ESM.docx]

# Appendix 2. Final WALK-Cph intervention.

| **Component** | **Intervention** | **Design** |
| --- | --- | --- |
| Welcome folder | On admission, health care professionals hand out a welcome folder, when introducing patients to the departments.  When handing out the welcome folder, health care professionals emphasize the importance of walking during and after hospitalization. | A WALK-Cph logo is printed on the front of the welcome folder (Department X)  The importance of walking during and after hospitalization is mentioned in the welcome folder. |
| WALK-path | Daily, the patients are motivated by all health care professionals to use the WALK-path for walking and exercising.  The patients are introduced to the WALK-path and the poster exercises by the nurses, nursing assistants, or the physiotherapists until the patients are self-reliant.  The patients should exercise using the WALK-path at least once a day. | The WALK-Cph project is assigned the color green (Department X) or blue (Department Y) and a pair of green or blue feet symbolizes the project. A WALK-path in the form of a line is stuck on the floor. In Department X, the line is marked with “WALK-path” and green feet. The WALK-path is rectangular, and a rest area is located at each length of the rectangle and marked with a green chair and a poster with three exercises. Furthermore, a small whiteboard is placed by the rest area for the patients to mark the number of rounds or minutes they have walked. It is the responsibility of the night staff to update the board on a daily basis (count the number of lines on the whiteboard.) The following is written on the board:  Yesterday’s rounds/minutes  Today’s rounds/minutes  In Department Y, the WALK-path is L-shaped, and a rest area is located at each length of the L and marked with blue chairs. |
| Posters with exercises placed in the wards | All health care professionals help motivate the patients to seek inspiration from the posters and perform the exercises by the rest areas of the WALK-path. | The posters by the WALK-path are identical to the posters in the wards. The posters show three exercises:  Walking: walk along the WALK-path  Balance: stand on one leg at a time (with a chair for aid)  Strength: stand up from a chair and sit down |
| Physician-prescribed WALK-plan | Daily, the physiotherapists and the nurses cooperate on evaluating all of the ward’s patients to decide which patients to prescribe a WALK-plan, the level of the WALK-plan, and possible changes to WALK-plan levels for those who have already been given a WALK-plan. The levels (colors) of all WALK-plan are noted on a patient board in the common office.  The physicians prescribe WALK-plans via the electronic patient record during rounds and motivate the patients to walk during the daytime. The physicians sign the WALK-plans before handing them out to the patients.  The WALK-plans are handed out to the patients by either the nurse or the physiotherapist.  The WALK-plans are noted in the rehabilitation plans (GOP) by the physiotherapist (only if the patient is discharged with a GOP).  The WALK-plan level is noted in the discharge reports by the nurse (if the patient receives help at home).  All groups of professionals have the responsibility to motivate the patients to walk and follow the WALK-plans. | Three different WALK-plans, with WALK-logos, are preprinted to limit the amount of time required to hand out the WALK-plans:  Level 1 (red): 1 minute – three times a day  Level 2 (yellow): 5 minutes – three times a day  Level 3 (green): 10 minutes – three times a day  At level 3, three exercises are suggested on the WALK-plan as a supplement. The exercises are identical to the exercises on the posters  The name of the patient is written on the plan |
| Independent collection of clothes* | The patients collect clothes from the wardrobes themselves. On admission, the health care staff introduce the patients to the wardrobes and motivate/follow the patients to collect clothes. | The wardrobes from which the patients may collect clothes are marked with WALK-logos. |
| Independent collection of beverages | The patients collect beverages from the beverage wagon and the refrigerator in the ward  On admission, the health care staff introduce the patients to the beverage wagon and the refrigerator and motivate the patients/follow the patients to collect beverages. |  |
| After discharge, patients with a WALK-plan, who are discharged with a rehabilitation plan, will be contacted by phone by a municipal therapist. | At discharge, the physiotherapist makes sure to note in the rehabilitation plan that the patient is discharged with a WALK-plan.  1–5 days after discharge, a therapist from the municipality contacts the patient by phone to motivate the patient to continue to follow the WALK-plan. |  |
| After discharge, patients with a WALK-plan, who are discharged without a rehabilitation plan, but receive home care, will be contacted by phone by home health care personnel. | At discharge, the nurse makes sure to note in the discharge papers for the municipality that the patient is discharged with a WALK-plan.  After discharge, when visiting the patient, the home care health care personnel motivate the patient to follow their WALK-plan. |  |

*This component was not a part of the intervention in Department Y.
